# Highly pathogenic avian influenza virus H5N1 controls type I IFN induction in chicken macrophage HD-11 cells: a polygenic trait that involves NS1 and the polymerase complex

**DOI:** 10.1186/1743-422X-9-7

**Published:** 2012-01-09

**Authors:** Matthias Liniger, Hervé R Moulin, Yoshihiro Sakoda, Nicolas Ruggli, Artur Summerfield

**Affiliations:** 1 Institute of Virology and Immunoprophylaxis (IVI), Sensemattstrasse 293, CH-3147 Mittelhäusern, Switzerland; 2 Department of Disease Control, Graduate School of Veterinary Medicine, Hokkaido University, Japan

**Keywords:** H5N1 avian influenza A virus, chicken HD-11 macrophage-like cell line, type I interferon, nonstructural protein 1, viral polymerase complex

## Abstract

**Background:**

Influenza A viruses are well characterized to antagonize type I IFN induction in infected mammalian cells. However, limited information is available for avian cells. It was hypothesised that avian influenza viruses (AIV) with distinct virulence may interact differently with the avian innate immune system. Therefore, the type I IFN responses induced by highly virulent and low virulent H5N1 AIV and reassortants thereof were analysed in chicken cells.

**Results:**

The highly pathogenic (HP) AIV A/chicken/Yamaguchi/7/04 (H5N1) (Yama) did not induce type I IFN in infected chicken HD-11 macrophage-like cells. This contrasted with an NS1 mutant Yama virus (Yama-NS1^A144V^) and with the attenuated H5N1 AIV A/duck/Hokkaido/Vac-1/04 (Vac) carrying the haemagglutinin (HA) of the Yama virus (Vac-Yama/HA), that both induced type I IFN in these cells. The substitution of the NS segment from Yama with that from Vac in the Yama backbone resulted in induction of type I IFN secretion in HD-11 cells. However, vice versa, the Yama NS segment did not prevent type I IFN induction by the Vac-Yama/HA virus. This was different with the PB1/PB2/PA segment reassortant Yama and Vac-Yama/HA viruses. Whereas the Yama virus with the Vac PB1/PB2/PA segments induced type I IFN in HD-11 cells, the Vac-Yama/HA virus with the Yama PB1/PB2/PA segments did not. As reported for mammalian cells, the expression of H5N1 PB2 inhibited the activation of the IFN-β promoter in chicken DF-1 fibroblast cells. Importantly, the Yama PB2 was more potent at inhibiting the IFN-β promoter than the Vac PB2.

**Conclusions:**

The present study demonstrates that the NS1 protein and the polymerase complex of the HPAIV Yama act in concert to antagonize chicken type I IFN secretion in HD-11 cells. PB2 alone can also exert a partial inhibitory effect on type I IFN induction. In conclusion, the control of type I IFN induction by H5N1 HPAIV represents a complex phenotype that involves a particular viral gene constellation rather than a single viral protein. Collectively, these findings contribute to understand the high virulence of HPAIV H5N1 viruses observed in the chicken host.

## Background

Type I interferons (IFN) exert key functions in the innate immune defence against influenza A virus infections by limiting viral spread and replication [1]. Host cells express a broad repertoire of pattern recognition receptors (PRRs) to viral danger signals. These include the membrane-bound Toll-like receptors (TLRs) and the cytoplasmic RIG-I-like receptors (RLRs) that sense unique viral structures such as single-stranded, double-stranded or 5'-triphosphorylated RNA [2]. In influenza A virus (IAV)-infected cells, the viral NS1 protein is involved in multiple regulatory functions, including the control of type I interferon (IFN) induction [3,4]. Although most of the studies focussed on the interaction of IAV with the type I IFN system in mammalian systems, several studies demonstrated also the critical role of NS1 in the pathogenesis of avian influenza viruses (AIV) in chicken. A recent study, for instance, reported that the highly pathogenic (HP) AIV A/goose/Guangdong/1/96 (H5N1) antagonized the induction of type I IFN in chicken embryo fibroblasts, whereas a recombinant virus carrying a valine instead of the alanine at position 149 of NS1 lost this function and became avirulent [5]. Another report demonstrated enhanced virulence related to a deletion of 5 amino acids in the NS1 protein at positions 80 to 84, typically observed in recently emerged HPAIV H5N1 isolates [6]. This deletion is located within the region that links the dsRNA binding domain and the effector domain. A study conducted in ducks reported that the exchange of the NS segments between a high and a low virulent H5N1 virus had a minimal impact on pathogenicity [7]. The authors therefore suggested other viral genes or combination of genes to be related to virulence. Mutations at multiple sites of PB2 contribute to the virulence and adaptation of H5N1 influenza in mice [8-10]. Only recently, the polymerase subunit PB2 was found to confer importin-α specificity and therefore to represent an important determinant of host range [11,12]. The viral polymerase complex was also found to decrease IFN-β induction in mammalian cells [13,14]. PB2 protein inhibits the transcription of the IFN-β mRNA by interacting with the RLR-adaptor CARDIF (also known as MAVS, IPS-1, VISA). Along this line, it was reported that exchanging the PB1, PB2 and NP segments alters viral replication of H5N1 reassortant viruses in chicken and can modulate pathogenicity [15]. Furthermore, the PB2 and NP of H5N1 HPAIV are associated with increased pathogenicity in chicken [16].

The HPAIV H5N1 A/chicken/Yamaguchi/7/04 (Yama) [17,18] induces a peracute disease with 100% mortality within 36 h in experimentally infected chickens [19,20]. Interestingly, a recombinant virus derived from the low pathogenic (LP) H5N1 virus A/duck/Hokkaido/Vac-1/04 (Vac) [21] and carrying the Yama HA segment (Vac-Yama/HA) is also virulent, resulting in 100% mortality in chicken, but with a delay of 48 h when compared with the Yama virus [20]. This latter study shows that type I IFN levels in lung, plasma and spleen are higher with the Yama virus than with the less virulent Vac-Yama/HA reassortant. Interestingly, infection with virulent AIV results in high viral load and severe disease in the presence of high type I IFN levels in the different organs. However, the impact of systemic type I IFN on disease pathogenesis during H5N1 infection of chicken remains elusive. We recently reported that chicken DF-1 fibroblasts and HD-11 macrophage-like cells employ MDA5 to sense AIV infections [22]. Based on these data, the present study was designed to identify viral factors controlling type I IFN induction in these two cell lines. To this end, the highly virulent H5N1 Yama and Vac-Yama/HA and multiple reassortants thereof were used to infect chicken HD-11 cells, DF-1 fibroblasts and splenocytes. With this *in vitro *study, viral genetic elements controlling type I IFN induction in H5N1 AIV-infected chicken cells were identified.

## Results

### HPAIV H5N1 as opposed to low pathogenic (LP) AIV do not induce type I IFN in embryonated chicken eggs

**Figure 1:**
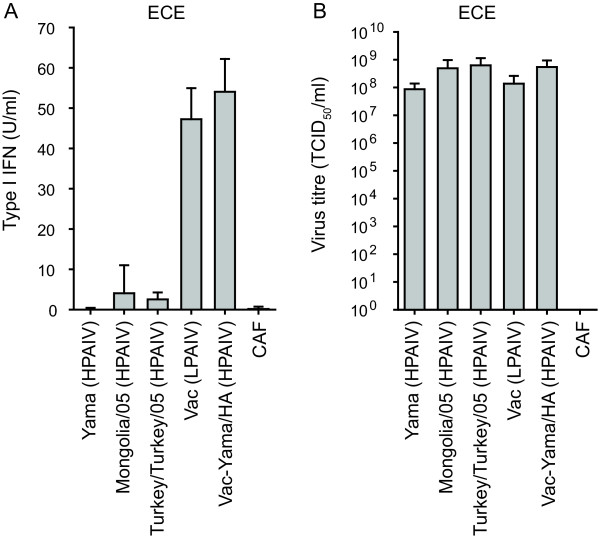
**Induction of type I IFN in H5N1 virus infected embryonated chicken eggs**. 10-day-old embryonated chicken eggs were inoculated with the indicated viruses with a dose of 10^3 ^TCID_50_/egg in 0.2 ml of PBS per egg. Allantoic fluid was harvested 24 h after infection and analysed for type I IFN bioactivity (A) and virus titre (B). Control allantoic fluid (CAF) was harvested from eggs inoculated with PBS. The data represent average values with standard deviations of 5 infected eggs.

This study employed multiple H5N1 AIV viruses (see table 1) generated by reverse genetics. These include the completely attenuated Vac and the highly virulent H5N1 Yama and A/whooper swan/Mongolia/3/05 viruses. In addition, the HPAIV field isolate A/turkey/Turkey/1/2005 H5N1 was used. A reassortant Vac-derived virus with the Yama HA segment (Vac-Yama/HA), and a Yama mutant carrying an NS1 A144V substitution (Yama-NS1^A144V^) corresponding to that described for A/goose/Guangdong/1/96 (H5N1) at position 149 [5] were generated. The Vac-Yama/HA was constructed to obtain a virus capable of replicating in the absence of trypsin. This was necessary due to the intolerance of HD-11 cells to trypsin. Additional segment exchange mutant viruses generated are listed in Table 1. Interestingly, during amplification of AIV in chicken eggs, some preparations contained antiviral activity. Therefore, type I IFN in the allantoic fluid was quantified systematically for all virus preparations. The three HPAIV H5N1 strains, including Yama, Mongolia-3/05, and Turkey/Turkey/05 did not induce any type I IFN in embryonated chicken eggs 24 h after infection (Figure 1A). These findings contrasted with the LPAIV Vac and the HPAIV Vac-Yama/HA virus that induced approximately 50 U/ml type I IFN in the infected eggs. Importantly, 1 day after infection when the allantoic fluids were harvested, the virus titres were comparable (Figure 1B). In order to avoid any carryover of chicken type I IFN with the virus stocks prepared in eggs, all viruses employed to infect chicken cells were produced in MDCK cells.

**Table 1:** H5N1 avian influenza A viruses employed in this study.

Name	Abbreviation or description	Pathogenicity in chicken, Reference
A/chicken/Yamaguchi/7/04	Yama	HP, [17]
A/duck/Hokkaido/Vac-1/04	Vac	LP, [21]
A/whooper swan/Mongolia/3/05	Mongolia/05	HP, [30]
A/turkey/Turkey/1/2005	Turkey/Turkey/05	HP, [31]
Yama-NS1^A144V^	Yama with mutant NS1 (A144V)	unknown
Yama-Vac/NS	Yama with Vac NS	unknown
Yama-Vac/PB2/PB1/PA	Yama with Vac PB2, PB1, PA	unknown
Yama-Vac/PB2/PB1/PA/NS	Yama with Vac PB2, PB1, PA and NS	unknown
Yama-Vac/PB1	Yama with Vac PB1	unknown
Yama-Vac/PB2	Yama with Vac PB2	unknown
Vac-Yama/HA	Vac with Yama HA	HP, [20]
Vac-Yama/HA/NS	Vac with Yama HA and NS	unknown
Vac-Yama/HA/PB2/PB1/PA	Vac with Yama HA, PB2, PB1, PA	unknown
Vac-Yama/HA/PB2/PB1/PA/NS	Vac with Yama HA, PB2, PB1, PA and NS	unknown
Vac-Yama/HA/PB1	Vac with Yama HA, PB1	unknown
Vac-Yama/HA/PB2	Vac with Yama HA, PB2	unknown

### The Yama virus does not induce type I IFN in HD-11 cells

**Figure 2:**
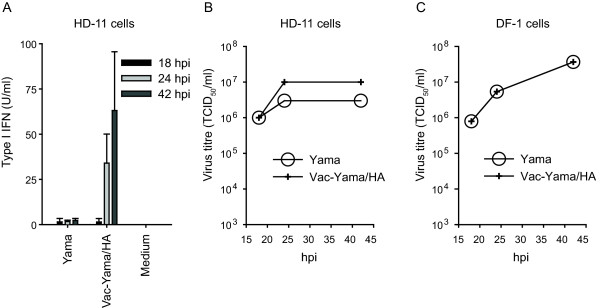
**The Yama virus controls type I IFN induction in HD-11 cells**. HD-11 and DF-1 cells were infected with Yama or Vac-Yama/HA virus at an MOI of 0.05 TCID_50_/cell. At the indicated hours post infection (hpi), cell culture supernatants were analysed for type I IFN bioactivity (A) and virus titres (B and C). For type I IFN, average values with standard deviations of triplicate cultures are shown. For the viral titers, pooled cell culture supernatants were analysed. The data are representative for two independent experiments.

In order to determine whether the observations made in eggs are also true in cell culture, the capacity of H5N1 viruses to induce type I IFN in chicken macrophage-like HD-11 cells and DF-1 fibroblast cells was investigated. As shown in Figure 2A, HD-11 cells produced type I IFN after infection with Vac-Yama/HA but not with Yama. This contrasted with DF-1 cells, where no type I IFN secretion was observed with any of these viruses (data not shown). However, the two viruses replicated to comparable levels in the two cell lines (Figure 2B and 2C). Relating to its inability to propagate in HD-11 cells, the trypsin-dependent LP Vac virus did not induce type I IFN in HD-11 cells nor in DF-1 cells (data not shown).

### The Yama virus induces type I IFN in chicken splenocytes

**Figure 3:**
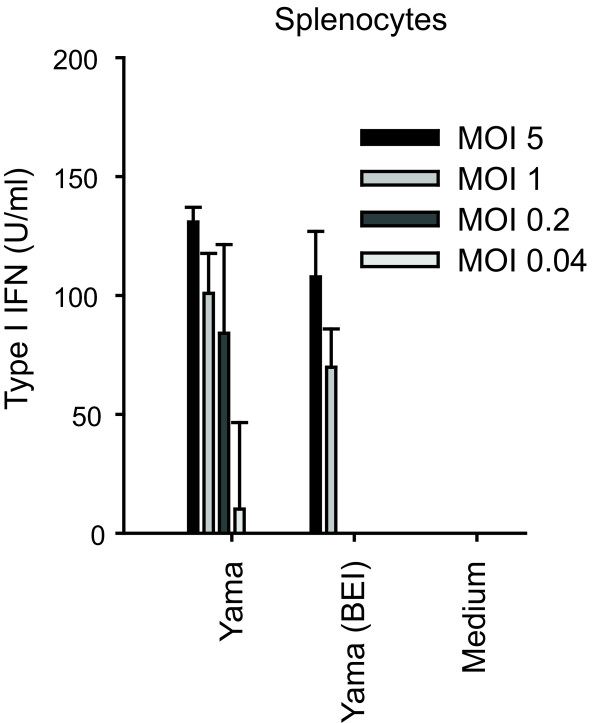
**The Yama virus induces type I IFN induction in chicken splenocytes**. Chicken splenocytes were stimulated with live and BEI-inactivated Yama virus at decreasing MOI. At 18 h after infection, culture supernatants were assayed for type I IFN bioactivity. Data are shown as average values of triplicate wells with standard deviations. The data are representative of two independent experiments.

Considering that HPAIV spreads to lymphoid tissue during infection in chicken, and that type I IFN responses are observed *in vivo *[19,20], the type I IFN responses to HPAIV infection were analysed in chicken splenocytes. Contrasting with HD-11 cells, the Yama virus induced type I IFN in infected splenocytes (Figure 3). Importantly, 2-bromoethylamine hydrobromide (BEI)-inactivated virus did also stimulate type I IFN production in splenocytes. BEI treatment abolished the infectivity completely, without affecting the capacity of the virus to haemagglutinate chicken red blood cells, indicating that the virion structure had maintained its integrity as shown earlier [23]. Altogether, these data indicate that Yama virus replication is not required for virus recognition and activation of type I IFN in chicken splenocytes.

### NS1 is required to prevent type I IFN induction in Yama-infected HD-11 cells

**Figure 4:**
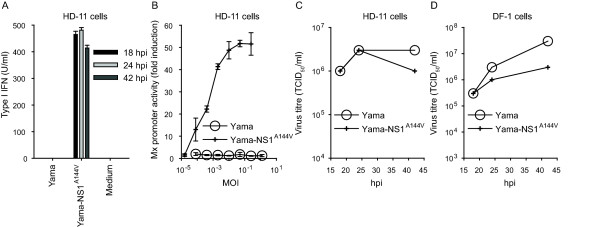
**The NS1 protein of Yama controls the induction of type I IFN in HD-11 cells**. HD-11 cells were infected with Yama or Yama-NS1^A144V ^at an MOI of 0.05 TCID_50_/cell (A). After 24 h, supernatants were tested for type I IFN bioactivity. The standard deviations of the type I IFN values were calculated from triplicate measurements of two cultures (A). The supernatants of HD-11 cells infected with increasing MOI of Yama or Yama-NS1^A144V ^were assayed for Mx-promoter induction in CEC-32 bioassay indicator cells (B). HD-11 (C) or DF-1 cells (D) were infected with the indicated viruses at an MOI of 0.05 TCID_50_/cell. At the indicated times after infection, virus titres were determined. The data are representative of two independent experiments.

Based on the known activity of NS1 as type I IFN antagonist [5,22], we assessed whether the viral NS1 protein is required to control type I IFN secretion in HPAIV-infected HD-11 cells. As opposed to the parental Yama strain, the NS1 mutant Yama-NS1^A144V ^induced high levels of type I IFN in HD-11 cells (Figure 4A). The induction was dependent on the virus dose (Figure 4B). Even at high multiplicity of infection (MOI), the Yama virus did not induce type I IFN in these cells. Surprisingly, despite the induction of type I IFN, the NS1 mutant virus, replicated to titres higher than 10^6 ^TCID_50_/ml in both, HD-11 and DF-1 cells (Figures 4C and 4D). These data demonstrate that the H5N1 Yama NS1 protein is required to prevent type I IFN secretion of infected HD-11 cells.

### The Yama NS segment in the Vac backbone is not sufficient to prevent type I IFN in HD-11 cells

**Figure 5:**
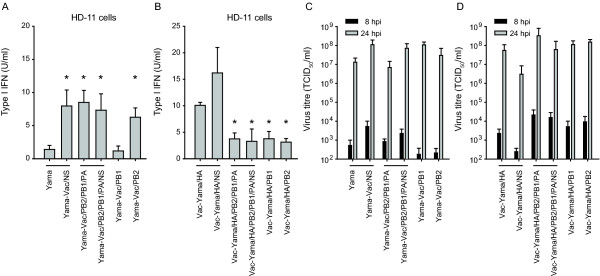
**Analysis of type I IFN responses and viral titres after infection of HD-11 cells with various segment-exchange mutant viruses**. HD-11 cells were infected with the indicated viruses at an MOI of 0.05 TCID_50_/cell. After 24 h the supernatants were analysed for type I IFN bioactivity (A and B). In parallel, corresponding virus titres were determined at 8 h and 24 h after infection (C and D). The data represent the mean values with standard deviations obtained from four independent experiments performed at least in triplicate. The asterisks (*) indicate statistical significant differences between selected viruses compared to Yama (A) or to Vac-Yama/HA (B) calculated with the students t-test (p < 0.05).

In order to determine the role of the NS segment in the different ability of the Yama and Vac-Yama/HA viruses to control type I IFN induction, we constructed NS segment-exchange mutants of the two viruses. Introduction of the Vac NS segment into the Yama virus (Yama-Vac/NS) disrupted the block of type I IFN induction (Figure 5A). However, introduction of Yama NS in the Vac-Yama/HA virus did not mediate block of type I IFN induction (Figure 5B). These results indicate that either the Yama NS1 requires additional viral determinants present only in the Yama genetic context, or that other viral determinants present only in the Yama virus contribute to type I IFN suppression.

### The polymerase complex of Yama is involved in the prevention of type I IFN induction in HD-11 cells

We next assessed the contribution of the polymerase complex in the control of type I IFN induction in virus infected HD-11 cells. Interestingly, the Yama virus with Vac-derived PB2/PB1/PA induced type I IFN, despite of the expression of Yama/NS1 (Figure 5A). Vice versa, the Vac-Yama/HA virus carrying all three Yama polymerase segments (PB2/PB1/PA) showed significantly reduced type I IFN induction, in comparison to Vac-Yama/HA (Figure 5B). Together, these results indicate that the control of type I IFN induction by the Yama virus in HD-11 cells represents a polygenic trait, involving the NS1 and the polymerase complex. As depicted in Figure 5C and 5D, all viruses grew to comparable titres, showing that the differences in type I IFN induction were not due to different levels of virus replication. Similarly, no differences in the kinetics of cell survival between the different reassortants were observed (data not shown).

### Yama PB2 plays a major role in the prevention of type I IFN induction in infected HD-11 cells

Next, reassortant Yama viruses carrying either the Vac PB1 or PB2 segments were analysed. The failure of Yama with Vac PB2 but not with Vac PB1 to control type I IFN, indicated a role of the Yama PB2 in type I IFN antagonistic activity of the virus (Figure 5A). The inhibitory effect of the Yama PB2 on the type I IFN induction pathway was confirmed with Vac-Yama/HA/PB2, as compared to Vac-Yama/HA (Figure 5B). Nevertheless, also Vac-Yama/HA/PB1 induced lower levels of type I IFN compared to Vac-Yama/HA, which may indicate redundant functions between PB1 and PB2 in inhibition of type I IFN induction (Figure 5B). In summary, our data demonstrate that the type I IFN induction in HD-11 cells is counteracted by the Yama polymerase complex with a prominent role of the PB2 gene segment.

### PB2 from the Yama virus exerts a stronger inhibitory activity on chicken type I IFN induction than PB2 from the Vac virus

**Figure 6:**
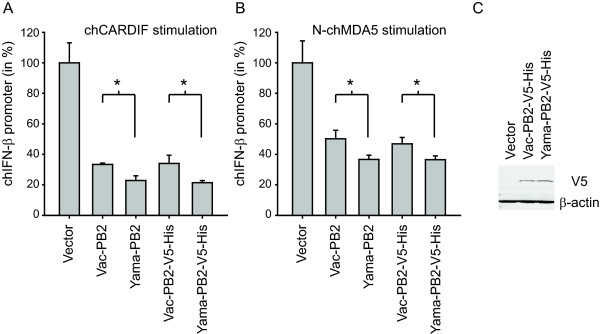
**Effect of PB2 expression on the chicken RLR-specific IFN-β promoter activation**. Chicken DF-1 fibroblast cells were transfected with a mixture of the chicken IFN-β promoter reporter plasmid, and of expression plasmids for untagged or V5- and His-tagged Vac-PB2 or Yama-PB2 (150 ng/well in A, 50 ng/well in B) and with expression plasmids for either chCARDIF (50 ng/well) or N-chMDA5 (12.5 ng/well) to trigger the chRLR signalling pathway. At 18 h after infection, the cell lysates were analysed using the dual luciferase assay. Data are shown as mean values with standard deviations obtained from six transfections. The asterisks (*) indicate statistical significant differences calculated with the students t-test (p < 0.05). The selected data are representative of two independent experiments. The expression of the V5-His-tagged Vac-PB2 and Yama-PB2 proteins in transfected DF-1 cells was analysed by Western blotting using the anti-V5 MAb (C). The β-actin content was determined with the anti-β-actin MAb.

In mammalian cells, PB2 was also found to inhibit IFN-β induction besides NS1 [13,14]. Consequently, we examined the putative inhibitory effects of Yama and Vac PB2 expression on the activation of the chicken RIG-I-like receptor pathway using a transient IFN-β promoter reporter assay in chicken DF-1 fibroblast cells. For this purpose, the IFN-β promoter was activated by expression of the chicken (ch) CARDIF and N-terminal domain of chMDA5 (N-chMDA5). Expression of the Vac or Yama PB2 proteins (with or without V5-His tag) decreased the IFN-β promoter activation triggered by chCARDIF and N-chMDA5 expression (Figure 6A and 6B). This was not seen with the M1 and PB1-F2 proteins from any of the two viruses (data not shown). In comparison to the Vac-derived PB2, expression of the Yama PB2 resulted in significantly stronger inhibition of RLR-specific chicken IFN-β promoter activation. With the tagged proteins, comparable levels of protein expression were demonstrated (Figure 6C). Interestingly, the Vac PB2 and Yama PB2 amino acid sequences differ only by four amino acids (M64I, I67V, T108A and K339T).

## Discussion

The present study revealed that type I IFN production in infected embryonated chicken eggs and HD-11 cells was related to the virulence of the virus. In particular, infection of HD-11 cells with the HP Yama virus did not result in type I IFN secretion, as opposed to infection with the less virulent Vac-Yama/HA virus. The viral factors determining type I IFN production were identified with mutational analyses, revealing the involvement of NS1 and the PB2 proteins of the Yama virus in the prevention of type IFN induction.

The fact that the A144V mutation within NS1 of the Yama virus results in strong induction of type I IFN indicates that NS1 functions as an inhibitor of the Yama virus-mediated type I IFN induction. A critical role of alanine 144 of H5N1 NS1 in the counteraction of type I IFN induction was also reported for the corresponding alanine at position 149 of the A/goose/Guangdong/1/96 H5N1 NS1 protein [5]. These data are in agreement with earlier results showing that type I IFN induction triggered by MDA5 in chicken cells can be inhibited by NS1 [22]. Interestingly and in apparent contradiction with the situation in HD-11 cells, stimulation of chicken splenocytes with live and with BEI-inactivated Yama virus resulted in considerable type I IFN secretion. Also in experimentally infected chicken, the Yama virus induces high systemic levels of type I IFN [19,20]. This may be explained by the interaction of AIV with specialized type I IFN producing cells present in splenocyte preparations and *in vivo*. These cells may be analogous to the mammalian plasmacytoid dendritic cells (pDCs), supported by the observation that their activation was independent of virus replication and therefore of NS1 expression, as it is the case for mammalian pDCs.

In order to elaborate on the gene segments that may be involved in the inhibition of type I IFN induction by the Yama virus, various reassortants of the Yama and Vac-Yama/HA viruses were analysed for type I IFN induction in HD-11 cells. The fact that the Vac NS segment in the Yama backbone abrogated the capacity of the virus to prevent type I IFN induction, but not vice versa, indicated that additional viral determinants were involved in the control of type I IFN induction. As expected from previous results with mammalian cells [13,14], we demonstrate a role of the polymerase complex, in particular of PB2, in the prevention of type I IFN induction in chicken cells. The effect mediated by PB2 could be measured in the absence of NS1 or other viral genes. However, the results suggest that the enhanced inhibitory activity exhibited by the PB2 protein of the Yama virus is likely to act in concert with NS1 and possibly other viral proteins. This relates to observations made with PB2 in mammalian cells [13,14]. The PB2 proteins may directly influence viral transcription and replication [16]. Alternatively, the PB2 proteins of the Yama and Vac viruses could interact differently with viral and cellular proteins such as nucleoproteins [24] or host-specific importin-α isoforms [11,12].

## Conclusions

The inhibition of type I IFN induction by HPAIV in HD-11 is a complex property that involves the NS1 and the PB2 of the viral polymerase complex. This inhibition is restricted to particular cell types. From these data one may speculate that the capacity of the HP Yama virus as opposed to the LP Vac virus to counteract early type I IFN induction at the primary replication sites *in vivo *contributes to the rapid establishment of infection and fast dissemination in the infected chicken host [19,25].

## Methods

### Cells

DF-1 chicken fibroblast cells (ATCC, LGC Standards, Molsheim, France) were propagated in DMEM + GlutaMAX-I (Invitrogen, Basel, Switzerland) in the presence of 10% heat-inactivated fetal bovine serum (FBS, Biowest) at 39°C and 5% CO_2_. The chicken macrophage-like cell line HD-11 (ATCC) [26] was cultivated in DMEM + GlutaMAX-I supplemented with 2% of chicken serum (Invitrogen) and 8% heat-inactivated FBS at 39°C with 5% CO_2_. The quail fibroblast cell line CEC-32 carrying a luciferase gene controlled by the chicken Mx promoter [27] was kindly provided by Dr Peter Staeheli, University of Freiburg, Germany. CEC-32 cells were kept in DMEM + GlutaMAX-I supplemented with heat-inactivated 8% FBS and 2% chicken serum at 39°C and 5% CO_2_. Human embryonic kidney (HEK) 293T cells (ATCC) were cultured in DMEM + GlutaMAX-I supplemented with 10% FBS at 37°C with 5% CO_2_. Madin-Darby canine kidney (MDCK) cells were grown in minimal essential medium (Invitrogen) supplemented with 10% fetal bovine serum (FBS), nonessential amino acids (Invitrogen), and 1 mM sodium pyruvate (Invitrogen) at 37°C with 5% CO_2_. Chicken splenocytes were isolated by cutting spleen into small pieces and disrupting cell aggregates through a 40 μm cell strainer (Becton Dickinson) followed by purification of the cells using Ficoll gradient centrifugation (1.077g/ml, GE Healthcare; 600 × g, 20 min). After two washes with PBS (250 × g, 10 min), the cells were cultured in DMEM, 10% FBS and 2% autologous chicken serum in 96-well plates at 2.5 × 10^5 ^cells/well, and stimulated with viruses for 24 h.

### Viruses

The viruses HPAIV H5N1 A/chicken/Yamaguchi/7/04 (Yama), HPAIV H5N1 A/whooper swan/Mongolia/3/05 and LPAIV H5N1 A/duck/Hokkaido/Vac-1/04 (Vac) were generated by reverse genetics using pHW2000-derived plasmids with the permission of Robert G. Webster (Department of Infectious Diseases, St. Jude Children's Research Hospital, Memphis, Tennessee, USA). The Vac virus is composed of the NA and NS gene segments from A/duck/Mongolia/47/01 (H7N1) and the PB2, PB1, PA, HA, NP, and M segments from A/duck/Mongolia/54/01 (H5N2) (for references see Table 1). The HPAIV A/turkey/Turkey/1/2005 (H5N1) isolate was kindly provided by Dr. William Dundon, IZSV Instituto Zooprofilattico Sperimentale delle Venezie, Venice, Italy. For the rescue of infectious viruses, co-cultures of HEK 293T and MDCK cells were transfected with the sets of eight pHW2000-derived plasmids essentially as described elsewhere [28,29]. Briefly, HEK 293T and MDCK cells were seeded in 2 ml of Opti-MEM I + GlutaMAX-I medium (Invitrogen), incubated overnight, and transfected with 1 μl TransIT-293 per 1 μg plasmid mixture as described by the manufacturer (Mirus). One day post transfection, the medium was replaced with Opti-MEM I + GlutaMAX-I containing 1 μg/ml of irradiated TPCK-trypsin (Worthington). Cell culture supernatants were harvested three days later and viruses were propagated in embryonated chicken eggs as described previously [29]. Alternatively, the viruses were propagated in MDCK cells. To this end, confluent MDCK cells were infected at a MOI of 0.1 using Opti-MEM I medium containing 1 ug/ml irradiated TPCK-trypsin. Virus containing supernatants were harvested 36 to 42 h after infection. The virus titre was determined by endpoint dilution in MDCK cells in the presence of 1 μg/ml TPCK-trypsin, and expressed as 50% tissue culture infectious doses (TCID_50_)/ml according to the Reed-Muench formula (WHO Manual on Animal Influenza Diagnosis and Surveillance). For particular experiments, virus was inactivated chemically using 2-bromoethylamine hydrobromide as previously described [29].

### Site-directed mutagenesis of NS1

The alanine to valine substitution at the amino acid position 144 of the Yama NS1 protein was introduced with PCR-based site-directed mutagenesis using the 5' phosphorylated oligonucleotides Y-NS1mutAV-R (5'-TCCTTCTTCTGTGAAAACTCTAAG-3') and Y-NS1mutAV-F (5'-GCAATCGTGGGAGAAATCTCAC-3'). The PCR was performed according to standard protocols using the pHW2000-derived plasmid carrying the Yama NS segment as template and the Accuprime Pfx polymerase (Invitrogen). The resulting DNA fragment was religated, and subsequent plasmid DNA was purified from transformed *E. coli*. The nucleotide sequence of the mutated NS1 segment was verified by automated DNA sequencing using the ABI 3130 Genetic Analyzer (Life Technologies).

### AIV infection of cells

HD-11 macrophages and DF-1 fibroblasts were seeded in 24-well cell culture plates at a density of 5 × 10^5 ^cells per well. The next day, the cells were infected at a MOI of 0.05 TCID_50_/cell with selected viruses produced on MDCK cells. Chicken splenocytes were seeded in 96-well cell culture plates at a density of 2.5 × 10^5 ^cells per well in 0.1 ml of DMEM + GlutaMAX-I supplemented with 16% FBS and 4% chicken serum. The splenocytes were immediately infected with 5-fold serial dilutions of virus in 0.1 ml Opti-MEM I + GlutaMAX-I. At different times after infection, the virus titre in the supernatant was determined in MDCK cells as described above, and type I IFN bioactivity was quantified with the bioassay described below. In order to monitor the survival of the HD-11 cells after infection, the cells were fixed and stained with a crystal violet solution containing 1% formaldehyde, 0.5% (w/v) crystal violet and 30% ethanol. The fixed and stained cells were washed extensively with tap water and treated with lysis solution composed of 0.6% SDS and 40 mM HCl in Isopropanol. The optical density of the crystal violet was measured at 595 nm.

### SDS-PAGE and Western blot analysis

Cells were lysed in a hypotonic buffer containing 20 mM morpholinepropanesulfonic acid, 10 mM NaCl, 1.5 mM MgCl2, 1% Triton X-100 (pH 6.5), and protease inhibitor cocktail (Sigma). Proteins were separated by SDS-PAGE under reducing conditions and analysed by Western blotting (41) using anti-V5 (Invitrogen), anti-HaloTag (Promega), or anti- -actin (C4) antibodies (Santa Cruz) and IRDye-labeled secondary antibodies (Li-Cor Biosciences). The signals were acquired and quantified using the Odyssey infrared imaging system (Li-Cor Biosciences).

### Chicken type I IFN bioassay in CEC-32 cells and transient chicken IFN-β promoter assay in DF-1 cells

The type I IFN bioactivity in the supernatants of stimulated cells was measured with an Mx promoter-driven luciferase reporter bioassay described elsewhere [27] and adapted for the 96-well plate format. Briefly, CEC-32 cells were seeded in 96-well plates at a density of 2 × 10^4 ^cells per well. The next day, dilutions of recombinant chicken IFN-α (kindly provided by Dr. Peter Staeheli, Freiburg, Germany) and virus-inactivated samples were added to the CEC-32 cells. The samples containing infectious virus were inactivated at 65°C for 30 minutes prior to the addition to the CEC-32 cells. Chicken IFN-α was resistant to this treatment. After 6 h of incubation at 39°C with 5% CO_2_, the CEC-32 cells were lysed and luciferase activity was determined using the Centro LB 960 luminometer (Berthold technologies). The data were expressed as type I IFN units per ml of sample. For the transient chicken IFN-β promoter reporter assay, DF-1 cells seeded at a density of 2-4 × 10^4 ^cells/well in a 96-well plate were co-transfected with 25-50 ng/well of the reporter plasmid pGL3-P-chIFN-β-luc expressing firefly luciferase under the control of the chicken IFN-β promoter [22] and with 0.1 ng/well of the plasmid phRL-SV40 (Promega) for constitutive expression of Renilla luciferase, using Fugene HD (Roche). Where indicated, plasmids for cytomegalovirus promoter driven expression of chCARDIF and N-chMDA5 were used to induce the IFN-β promoter-mediated luciferase expression [22]. The cells were lysed at different times after stimulation using 20 μl of 1× passive lysis buffer (Promega). The samples were assayed for firefly and Renilla luciferase activity using the Dual-Luciferase Reporter Assay System (Promega) and the Centro LB 960 luminometer (Berthold Technologies). The firefly luciferase activity was normalized with the corresponding Renilla luciferase activity.

## Competing interests

The authors declare that they have no competing interests.

## Authors' contributions

Conceived and designed the experiments: ML, NR, AS, YS. Performed the experiments: ML, HM. Analysed the data: ML, NR. Wrote the paper: ML, NR, AS, YS.

All authors have read and approved the final manuscript.
